# Pneumothorax Following Breast Surgery at an Ambulatory Surgery Center

**DOI:** 10.7759/cureus.24924

**Published:** 2022-05-11

**Authors:** David N Flynn, Jenny Eskildsen, Jacob L Levene, Jennifer D Allan, Ty L Bullard, Kathryn W Cobb

**Affiliations:** 1 Department of Anesthesiology, University of North Carolina School of Medicine, Chapel Hill, USA

**Keywords:** iatrogenic complication, ambulatory surgery center, ambulatory anesthesiology, mastectomy, pneumothorax

## Abstract

Pneumothorax is a known complication following breast surgery but is likely underappreciated by anesthesiologists. Iatrogenic pneumothorax can be caused by needle injury during local anesthetic injection, surgical damage to the intercostal fascia or pleura, or pulmonary injury from mechanical ventilation. We present two cases of pneumothorax following bilateral mastectomy with bilateral pectoral blocks and immediate breast reconstruction. Both cases occurred at a freestanding ambulatory surgery center in patients with no history of lung disease. One patient was found to have bilateral pneumothoraxes after complaining of shortness of breath and chest pain in the post-operative care unit. The second patient was asymptomatic but found to have a right-sided pneumothorax on a chest X-ray (CXR) that was ordered to rule-out left-sided pneumothorax due to concern of intraoperative breach of the left chest wall. Both patients were treated with chest tubes, transferred to a nearby hospital, and discharged several days later. Anesthesiologists must be aware of this potentially life-threatening complication and consider pneumothorax in the differential diagnosis of perioperative hypoxemia, shortness of breath, chest pain, and hemodynamic collapse in patients undergoing breast surgery. Though traditionally diagnosed via radiograph, pneumothorax can be rapidly diagnosed with ultrasound. Tension pneumothorax should be decompressed immediately with a needle. A clinically significant, non-tension pneumothorax is treated with chest tube placement. Equipment necessary to treat pneumothorax should be available for emergency treatment in facilities wherever breast surgery is performed.

## Introduction

Pneumothorax is a known complication following breast surgery but is likely underappreciated by anesthesiologists [1-4]. Iatrogenic pneumothorax can be caused by needle injury during local anesthetic injection, surgical damage to the intercostal fascia or pleura, or pulmonary injury from mechanical ventilation. Pneumothorax can cause shortness of breath, tachypnea, chest pain, and hypoxia [5]. The development of tension pneumothorax is a life-threatening emergency that can rapidly precipitate cardiovascular collapse if left untreated [5].

The incidence of pneumothorax following surgeries involving the breast is unknown. Osborn JM and Stevenson TR surveyed California's plastic surgeons in 2001, with 50% of respondents reporting that they had operated on at least one patient who suffered a pneumothorax while undergoing breast augmentation [1]. Wickman M et al. reported a series of 30 patients who underwent prophylactic bilateral reconstruction with immediate reconstruction, noting one patient with a history of severe asthma who developed a pneumothorax [6]. Isaksson K et al. reported a cohort of 185 patients who underwent bilateral mastectomies with implant-based immediate reconstruction, noting that two patients developed pneumothorax [7]. Gandamihardja TA et al. reported that three out of 749 patients developed pneumothorax following mastectomy with immediate extended latissimus dorsi flap. One of the three patients developed a tension pneumothorax which required immediate needle decompression [3].

We present two cases of pneumothorax following bilateral mastectomy with bilateral pectoral blocks and immediate breast reconstruction. Both cases occurred at a freestanding ambulatory surgery center in patients with no history of lung disease. We review potential causes, diagnosis, and treatment of pneumothorax, and discuss the importance of pneumothorax vigilance and preparedness at ambulatory surgery centers.

## Case presentation

Case 1

The patient was a 29-year-old, 75-kgs woman with a history of deleterious BRCA2 mutation. She had no other relevant medical or surgical history. She was scheduled for a risk-reducing bilateral mastectomy with immediate reconstruction with pre-pectoral tissue expanders.

The patient was premedicated with midazolam in the preoperative area. General anesthesia was induced with IV lidocaine, propofol, fentanyl, and succinylcholine. She was intubated via direct laryngoscopy with a 7.0 mm endotracheal tube. General anesthesia was maintained with sevoflurane (1.4-1.8% end-tidal concentration) and propofol infusion (50 mcg/kg/min). Lung protective ventilation was employed using pressure-controlled ventilation, with inspiratory pressures of 12 cm H20 achieving tidal volumes of approximately 8 ml/kg ideal body weight and positive-end expiratory pressure (PEEP) of 5 cm H20. Throughout the surgery, the patient had an oxygen saturation of 100% with an inspired oxygen concentration of 32-34%.

**Figure 1:**
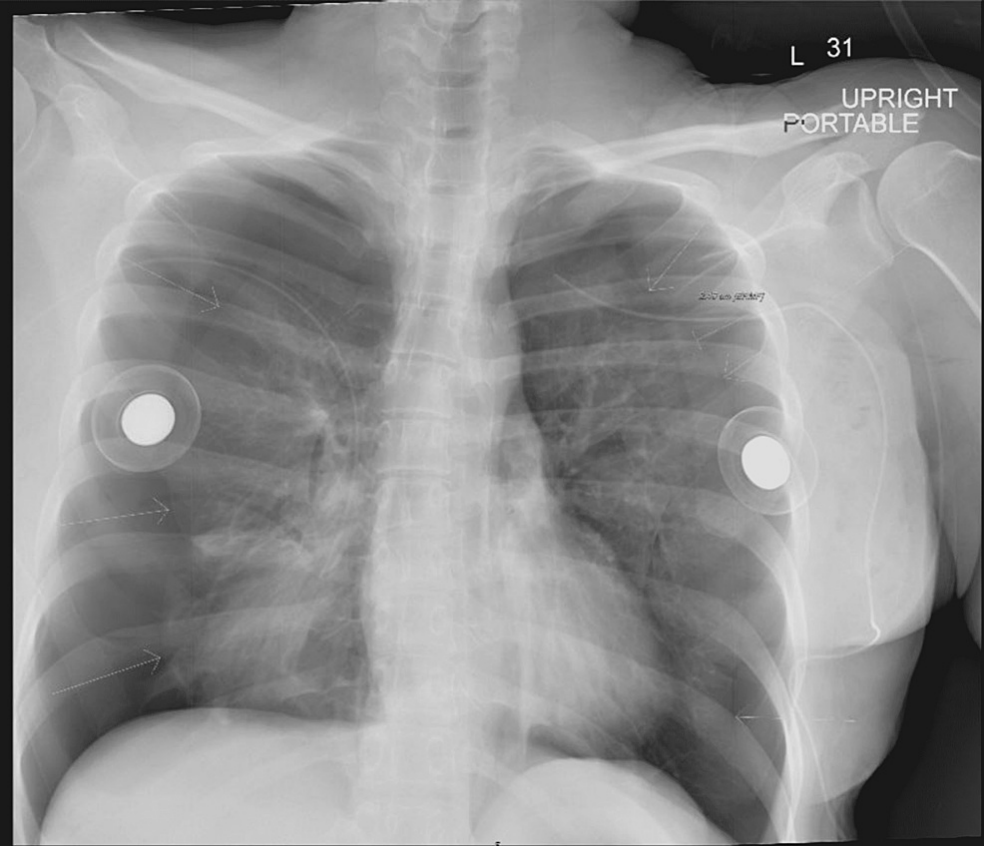
Bilateral pneumothoraxes following breast surgery.

Postoperatively, the patient complained of shortness of breath and chest pain. Her oxygen saturation was noted to be 91% despite administration of supplemental oxygen (6 liters per minute) via face mask. A chest X-ray (CXR) was obtained that demonstrated large bilateral pneumothoraxes (Figure 1). Subsequently, bilateral 24 Fr chest tubes were inserted between the fifth and sixth ribs at the mid-axillary line. The chest tubes were placed to suction. The patient was subsequently transferred to the nearby hospital. The chest tubes remained on suction on postoperative day 1 (POD1), were put to water seal on POD2, and were removed on POD3. She recovered fully and was discharged home on POD4.

Case 2

The patient was a 41-year-old, 72-kgs woman with stage 1B left-sided breast cancer. She had no other relevant medical or surgical history. Nevertheless, she was scheduled for a bilateral mastectomy with immediate reconstruction with pre-pectoral tissue expanders.

The patient was premedicated with midazolam in the preoperative area. General anesthesia was induced with IV lidocaine, propofol, fentanyl, and succinylcholine. She was intubated via direct laryngoscopy with a 7.0 mm endotracheal tube. General anesthesia was maintained with sevoflurane (1.2-1.7% end-tidal concentration) and propofol infusion (80-100 mcg/kg/min). The surgeon performed bilateral pectoralis blocks following completion of the mastectomies. Rocuronium was then given per the surgeon's request at the start of reconstruction. The surgeon noted a 1-2 mm chest wall defect during elevation of the left pectoralis minor muscle. This was repaired and tested by Valsalva maneuver, and with no evidence of ventilatory or hemodynamic compromise. A postoperative CXR was planned for further evaluation.

During the mastectomy portion of the procedure, mechanical ventilation was achieved with pressure support without PEEP, achieving tidal volumes of 6 ml/kg ideal body weight. After administration of rocuronium, the ventilatory mode was changed to pressure-controlled ventilation (9 cm H2O), with tidal volumes of 6-8 ml/kg ideal body weight and PEEP of 5 cm H2O. The patient had an oxygen saturation of 99-100% throughout the procedure, with an inspired oxygen concentration of approximately 60%. She was extubated to room air and taken to the post-anesthesia care unit (PACU).

In the PACU, the patient had well-controlled pain. She was breathing without difficulty, hemodynamically stable, and maintained an oxygen saturation near 100% without supplemental oxygen. The postoperative CXR demonstrated a large right-sided pneumothorax with a near-complete lung collapse, the opposite side in question during the surgery (Figure 1B). A 20 Fr chest tube was placed at the mid-axillary line, and the patient was transferred to the nearby hospital via ambulance. On POD2, the chest tube was removed, and she was discharged home on POD3.

**Figure 2:**
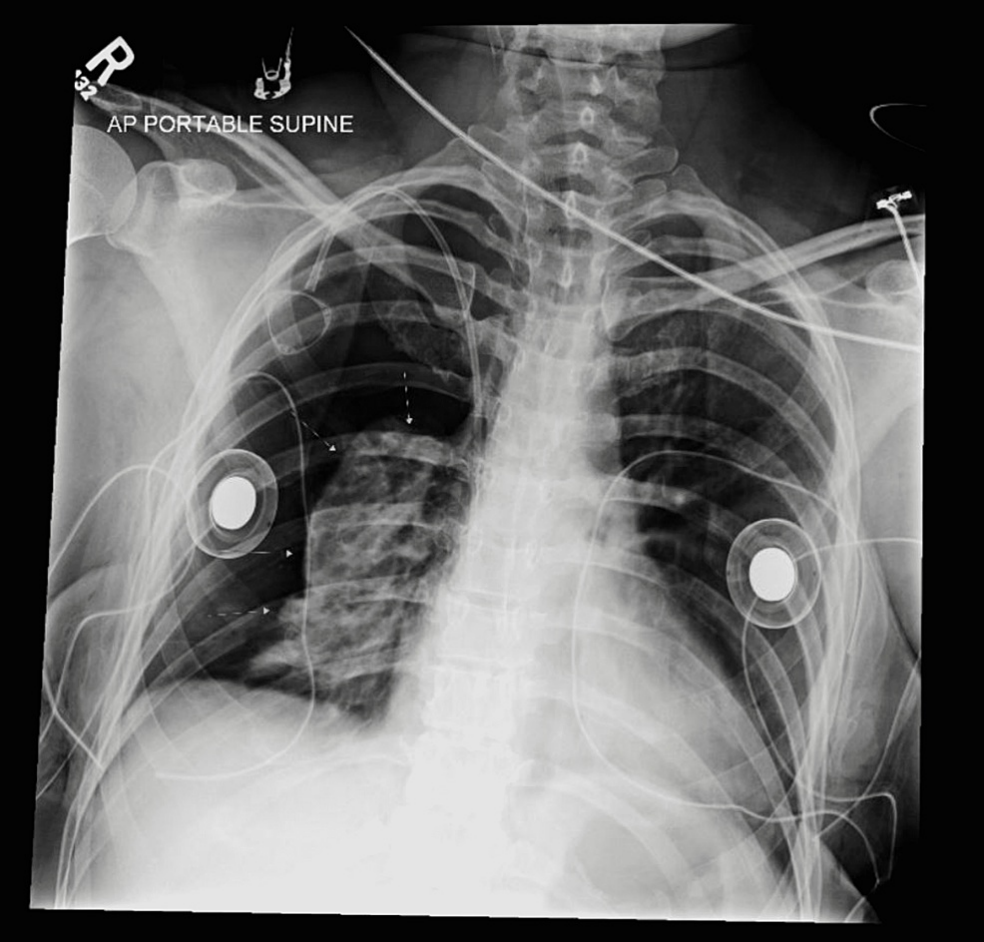
Right-sided pneumothorax with a nearly complete lung collapse in a patient following breast surgery.

## Discussion

Pneumothorax is a known complication following breast surgery, but the incidence is unknown. We report two cases of pneumothorax following bilateral mastectomy with immediate breast reconstruction. Both patients also had intraoperative pectoral blocks performed following mastectomy by the surgeon without ultrasound. In addition, one patient suffered bilateral pneumothoraxes, which, to our knowledge, has only been reported on one previous occasion [2]. Interestingly, there was a concern for a left-sided pneumothorax with the second patient due to a breach of the left chest wall during dissection. However, an unexpected right-sided pneumothorax was discovered, demonstrating near-total lung collapse.

Neither patient had a history of lung disease or connective tissue disorder. Both were intubated uneventfully, and there were no ventilatory volumes or pressures that would be expected to cause lung injury. Endotracheal intubation was used due to the long duration of these cases, which often take four hours or longer. During the surgical procedure or pectoral blocks, no damage to the intercostal fascia or pleura on the affected hemithoraces. However, the needle tip could not be visualized during the blocks since ultrasound was not used. There were no intraoperative changes in either patient's vital signs, ventilatory parameters, or oxygen requirements that triggered suspicion of pneumothorax during either surgery. Although the patient in case 1 exhibited classic signs of pneumothorax, including chest pain, shortness of breath, and hypoxia, the patient in case 2 was asymptomatic despite the near-total collapse of the affected lung. It is possible that this pneumothorax would not have been discovered if a CXR had not been performed to rule out pneumothorax on the opposite side.

It is possible that the needle tip caused the pneumothoraxes during the intraoperative pectoral blockade. The surgeon's performance of the block during surgery allows for excellent visualization and differentiation of the relevant muscle layers following surgical dissection. However, once the block needle is inserted into the muscle, the tip is no longer visible, and inadvertent puncture of the pleura is possible. Therefore, the performance of the pectoral blocks under ultrasound guidance may reduce the risk of pneumothorax, as long as the needle tip is maintained within view to ensure avoidance of the pleura. 

Anesthesiologists must have a high index of suspicion and consider pneumothorax when patients undergoing breast procedures develop chest pain, shortness of breath, tachypnea, hypoxia, hypotension, or cardiovascular collapse during the perioperative period. Physical exam findings consistent with pneumothorax include dyspnea; diminished, absent, or unequal breath sounds; hyperresonance to percussion; and crepitus [5,8]. Additionally, patients with tension pneumothorax may exhibit jugular venous distention and signs of shock due to reduced venous return to the heart [5,8]. Tension pneumothorax is a medical emergency that requires immediate treatment via needle decompression or thoracostomy.

Diagnosis of pneumothorax can be confirmed with diagnostic imaging. Ultrasound is readily available in many centers that perform surgical procedures. Ultrasound is an excellent diagnostic tool for pneumothorax, with a sensitivity of 91% and specificity of 99% [9]. Ultrasound signs that support the presence of pneumothorax include lack of lung sliding and lung pulse, absent B-lines, and identification of a lung point [10]. On CXR, evidence of pneumothorax includes visualization of a visceral pleural line and absent lung markings beyond the pleural line. A pneumothorax is classified as large when the distance between the chest wall and the pleural line is 2 cm or greater [11].

Anesthesiologists must understand how to treat a patient with a pneumothorax and ensure that necessary equipment and supplies are readily available. Observation with close follow-up is reasonable for an asymptomatic patient with small pneumothorax. Patients who are symptomatic or have a moderate or large pneumothorax require chest tube placement. The most recent guidelines from the American College of Surgeons Advanced Trauma Life Support (ATLS) recommend using smaller (28-32 Fr) chest tubes to treat pneumothorax [8]. However, smaller tubes, including pigtail catheters, may be equally effective [12]. Tension pneumothorax requires immediate decompression. Traditionally, needle decompression was recommended within the second intercostal space at the midclavicular line. However, most recent guidelines recommend accessing the fourth or fifth intercostal space at the mid-axillary line on the affected side [5,8].

It is important to maintain the equipment and supplies necessary to treat pneumothorax within surgical facilities that perform breast surgery. Recommended equipment includes chest tube insertion trays, thoracostomy tubes, catheters, and a chest tube drainage system.

## Conclusions

Pneumothorax is a complication following breast surgery, but the incidence is unknown. Anesthesiologists must be aware of this complication and consider pneumothorax in patients with hypoxia, shortness of breath, chest pain, or cardiovascular collapse. Clinicians who care for patients undergoing breast surgery must understand how to diagnose and treat pneumothorax. Facilities, where breast surgeries are performed, must maintain equipment and supplies so that clinicians can rapidly treat pneumothorax when it occurs.
